# Tracking Protein Motions using Serial Femtosecond Crystallography with X‐Ray Free‐Electron Laser

**DOI:** 10.1002/cpz1.70212

**Published:** 2025-09-23

**Authors:** Eiichi Mizohata, Eriko Nango, Takehiko Tosha, So Iwata, Minoru Kubo

**Affiliations:** ^1^ Graduate School of Engineering Osaka University Osaka Japan; ^2^ Institute of Multidisciplinary Research for Advanced Materials Tohoku University Miyagi Japan; ^3^ Graduate School of Science University of Hyogo Hyogo Japan; ^4^ Graduate School of Medicine Kyoto University Kyoto Japan

**Keywords:** molecular movie, protein motion, SACLA, structure–function relationship, time‐resolved analysis

## Abstract

Since the birth of biochemistry, researchers have investigated the structure–function relationship of a wide variety of proteins. However, until recently, when X‐ray free‐electron lasers (XFELs) became available, it was not possible to visualize the motion of proteins from moment to moment with excellent temporal and spatial resolution. Here, we introduce practical methods to visualize protein motions at room temperature using serial femtosecond crystallography (SFX) using XFELs. With the development of this technology, it will be possible to visualize the entire reaction mechanism of many proteins in the future. We first outline a streamlined microcrystallization workflow for hen egg‐white lysozyme, enabling rapid detector calibration and data‐collection optimization. Next, we present a rotational seeding approach refined on copper‐containing nitrite reductase that yields homogeneous microcrystals suitable for high‐resolution SFX and readily adaptable to other challenging targets. Finally, we describe a time‐resolved strategy combining microcrystals of fungal nitric‐oxide reductase with photolabile caged substrates and synchronized UV triggering, capturing catalytic intermediates on the millisecond timescale. Together, these procedures enable investigators to progress from preparing samples to capturing dynamic structural snapshots. © 2025 The Author(s). Current Protocols published by Wiley Periodicals LLC.

**Basic Protocol 1**: Microcrystallization of lysozyme

**Basic Protocol 2**: Microcrystallization of copper‐containing nitrite reductase

**Basic Protocol 3**: Time‐resolved serial femtosecond crystallography

## Introduction

Single crystal X‐ray crystallography is the most powerful and reliable technology that can resolve three‐dimensional protein structures with (near) atomic resolutions (0.5–3.9 Å) and has been at the frontier of structural biology for many decades. Traditionally, determining crystal structures at high resolution requires preparation of large crystals (>200 µm) to collect diffraction data using in‐house diffractometers. The construction of synchrotron radiation facilities that produce strong X‐rays has enabled structure determination at excellent resolutions even with small crystals (200–50 µm). Advances in radiation facilities and beamline technology have accelerated the determination of protein structures. The trend will continue with the development of various fourth‐generation light sources. One of the most important radiation technology innovations in recent years is the development of the X‐ray free‐electron laser (XFEL), which has the unique features of extreme peak brightness, ultrashort duration, and high spatial coherence of X‐ray pulses. Following the construction of the world's first XFEL facility, the Linac Coherent Light Source (LCLS) in the United States, in 2009 (Emma et al., 2010), the SPring‐8 Angstrom Compact Free‐Electron Laser (SACLA) was constructed in Japan in 2011 (Ishikawa et al., 2012). Subsequently, the European XFEL in Germany, the Pohang Accelerator Laboratory (PAL)‐XFEL in Korea, and the SwissFEL in Switzerland were built (Decking et al., 2020; Kang et al., 2017; Prat et al., 2020).

In this protocol, we describe practical methods of serial femtosecond crystallography (SFX), a new method for X‐ray crystal structure analysis that takes advantage of the unique features of XFEL (Chapman et al., 2011). SFX measurements enable data collection from many randomly oriented microcrystals (50–1 µm) streamed with an injector device across the XFEL beam, which allows single‐pulse diffraction from a microcrystal within femtosecond exposure times at room temperature (Sugahara et al., 2015; Tono et al., 2015). Because XFEL is 1 billion times brighter than conventional synchrotron radiation, the diffraction intensity obtained with femtosecond exposure of an XFEL pulse is comparable to the diffraction intensity with exposure of synchrotron radiation for 1 s. The time required for data collection depends on the condition of the sample. Typically, collecting diffraction images from thousands to half a million microcrystals requires an hour to several days.

Since conventional synchrotron radiation crystallography (SRX) requires exposure times of at least a millisecond or more for data collection, radiation damage and X‐ray photoreduction of the sample cannot be ignored; the interactions between X‐rays and water molecules in the crystal generate hydrated electrons on the order of picoseconds and these species react with protein molecules to break chemical bonds and reduce metal active centers. In contrast, SFX allows damage‐ and photoreduction‐free structural analysis of the sample (Fukuda, Tse, Nakane et al., 2016; Fukuda, Tse, Suzuki et al., 2016) because the XFEL diffraction process occurs on a timescale shorter than that needed for the generation of hydrated electrons. Moreover, the most important feature of SFX is that it can be applied to time‐resolved analyses (Kern et al., 2018; Nango et al., 2016; Suga et al., 2017; Tenboer et al., 2014; Tosha et al., 2017). Time‐resolved SFX (TR‐SFX) is the only measurement method that can visualize protein motions during functional activity (e.g., catalysis) at near‐atomic resolution and under ambient temperature conditions.

The methods described below comprise three protocols essential for conducting successful serial SFX experiments, as established in the SACLA‐SFX Project (Mizohata et al., 2018). Basic Protocol 1 details the methods for microcrystallization of lysozyme, a standard reference protein commonly utilized in initial SFX trials to optimize the detector geometry and experimental setup (Sugahara et al., 2015). This protocol includes the preparation of necessary solutions, crystallization conditions, and procedures for measuring crystal density. Basic Protocol 2 describes microcrystallization methods developed using copper‐containing nitrite reductase (CuNiR) as a model enzyme, employing a rotational seed crystallization technique that enables homogeneous microcrystal production, essential for advanced structural studies and visualization of catalytic processes (Fukuda, Tse, Nakane et al., 2016; Fukuda, Tse, Suzuki et al., 2016). Basic Protocol 3 provides comprehensive instructions for performing TR‐SFX, utilizing microcrystals of fungal nitric oxide reductase (P450nor) combined with photo‐sensitive caged compounds (Tosha et al., 2017). This approach allows precise observation and characterization of transient reaction intermediates, providing valuable insights into enzyme reaction dynamics.

*CAUTION*: All work involving recombinant DNA, microorganisms, hazardous chemicals, high‐power lasers, pressurized injectors, and intense X‐ray pulses must be carried out in full compliance with all applicable regulations and with the safety policies of both the XFEL facility and the participating institutions. All experimental protocols must receive prior approval from the facility's Safety Review Committee and the relevant institutional biosafety and environmental health and safety committees.

## Basic Protocol 1 MICROCRYSTALLIZATION OF LYSOZYME

In this section, we introduce methods for preparing lysozyme microcrystals, which are often used as a standard sample for the detector geometry refinement in SFX experiments. When performing SFX experiments for the first time, we recommend carrying out preliminary SFX experiments with lysozyme before using the target protein of interest. This ensures a better understanding of the methods of microcrystallization and crystal density measurements.

### Materials

Sodium acetate trihydrate (Wako Pure Chemical Industries, 198‐01055)
Acetic acid (Wako Pure Chemical Industries, 017‐00256)
Sodium chloride (Wako Pure Chemical Industries, 191‐01665)
PEG 6000, 50% (w/v) (Hampton Research, HR 2‐533)
Lysozyme (egg white) (Wako Junyaku, 120‐02674)

pH meter
Graduated beakers
Filters, 0.22 µm
Centrifuge tubes, 50 ml (Falcon)
Thermomixer C (Eppendorf)
Eppendorf SmartBlock, 50 ml (Eppendorf)
High‐performance microscope with a magnification of not less than 1500 (Hirox KH‐8700)
Refrigerated centrifuge
CellTrics filter, 30 µm (Sysmex)
Slide glass and cover glass
Cell counting plate, OneCell counter (Biomedical Science)
Mechanical Tally counter

#### Preparation of various solutions

This crystallization protocol is based on the method for obtaining micron‐sized crystals reported previously (Falkner et al., 2005). Whereas lysozyme typically crystallizes between pH 4 and 5, microcrystals of lysozyme can be produced with a precipitant buffer of pH 3.0. Crystals form instantaneously when a precipitant and a lysozyme solution are mixed. It is possible to vary the size of the crystal by changing the temperature (Nango et al., 2015). Lysozyme tends to form smaller crystals at lower temperatures. Here, we will explain how to prepare 5‐µm lysozyme microcrystals by fixing the temperature at 17°C.

1 Buffer A (1 M sodium acetate buffer, pH 3.0): Add ∼2.5 ml of 1 M sodium acetate to 100 ml of 1 M acetic acid as a guide and then adjust the solution to pH 3.0 with a calibrated pH meter. Ultrapure water should be used for all buffer preparations.
2 Crystallization solution [28% (w/v) sodium chloride, 8% (w/v) PEG 6000, and 0.1 M sodium acetate buffer, pH 3.0]: Add 10 ml of Buffer A to a graduated beaker. Add 28 g of sodium chloride and 16 ml of 50% (w/v) PEG 6000 to the beaker. Then add ultrapure water to the mixture as close to 100 ml as the final volume. Mix the solution with a stirrer for several hours to overnight. After fully dissolving the sodium chloride, dilute the solution with a small amount of ultrapure water to 100 ml and pass this solution through a 0.22‐µm filter. Store the buffer at room temperature to avoid salt precipitation, but do not store it for more than 1 week because of changes in pH.
3 Harvest solution for lysozyme microcrystals [10% (w/v) sodium chloride, 1 M acetate buffer (total acetate species = 1 M, pH 3.0)]: Add 10 g of sodium chloride and 5.75 ml of acetic acid to a beaker. Dilute the mixture with ultrapure water to about 80 ml. Adjust the solution to pH 3.0 with approximately 3 ml of 1 M sodium acetate solution (Note that this is not Buffer A in step 1). After filling with ultrapure water to 100 ml, pass the solution through a 0.22‐µm filter and store the harvest solution at 4 °C.
4 Lysozyme solution: Dilute Buffer A 10‐fold with ultrapure water to prepare 10 ml of 0.1 M sodium acetate buffer. Add 200 mg of lysozyme powder to 9 ml of the sodium acetate buffer on ice. Adjust the solution to a final concentration of 20 mg/ml lysozyme and pass the solution through a 0.22‐µm filter. Prepare this lysozyme solution when needed because the size of the crystals may change if left for more than 1 day.

#### Microcrystallization and recovery of crystals

**Figure 1:**
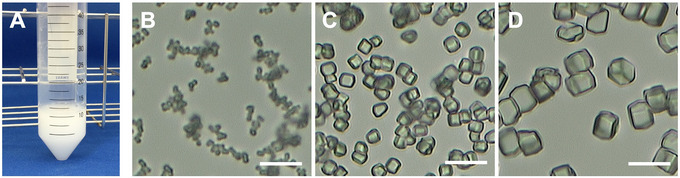
Microcrystallization of hen egg‐white lysozyme. (**A**) Appearance of lysozyme microcrystals immediately after preparation. Panels (**B**), (**C**), and (**D**) show crystals obtained by crystallization at 12°C, 17°C, and 21°C, resulting in long‐axis sizes of approximately 1 µm, 5 µm, and 10 µm, respectively. Scale bars represent 20 µm in all panels.

5 Add 10 ml of the lysozyme solution and 10 ml of the crystallization solution to two different 50‐ml centrifuge tubes.
6 Place these 50‐ml tubes in a Thermomixer C that is set at 17°C and stir for 30 min at 500 rpm. The thermomixer can be rotated and shaken in the horizontal direction while controlling the temperature. The mixer temperature should be set to 17°C to ensure microcrystals are 5 µm in size (set to 12°C or 21°C if the size should be 1 or 10 µm, respectively).
7 After 30 min, take the crystallization solution from the thermomixer (paying attention not to heat the crystallization solution by hand) and quickly add it to the lysozyme solution in one stroke and stir this solution using the thermomixer for at least 10 min. During this step, the lysozyme solution becomes white and turbid after the addition of the crystallization solution, and homogeneous microcrystals with a long side of approximately 5 µm are generated (Fig. 1).

8 After confirming microcrystal formation with a high‐performance microscope, centrifuge the microcrystalline solution 5 min at 3000 × *g*, 4°C. Decant the supernatant, add 10 ml of the harvest solution to the crystals, and suspend by inversion.
9 Pass the microcrystal suspension through a 30‐µm CellTrics filter to remove larger crystals or precipitants. This step prevents clogging of the injector nozzle during SFX data collection.

#### Crystal density measurement and quality‐control checks

Crystal density and quality are very important for SFX data collection. If the density is less than 10^6^ crystals/ml, the probability of the X‐ray pulse hitting the crystal is low, and thus the data collection time will be long. If the density is much higher than 10^8^ crystals/ml, diffraction patterns from multiple crystals are recorded in one image and this causes a lower indexing rate. Therefore, measuring and adjusting the crystal density in advance is desirable.

10 Dilute the crystal solution 500‐fold with the harvest solution.
11 Load 10 µl of the microcrystal suspension into a cell counting plate.
12 Measure the size of crystals in the cell counting plate by observation under a high‐performance microscope and count the number of crystals with a Tally counter. Calculate the crystal density according to the procedure of the cell counting plate.
13 The described crystallization procedure should yield an approximate crystal density of 1–2 × 10^8^ crystals/ml.
14 If the crystal quality is poor (e.g., edges are indistinct or the crystals are aggregated), one possible countermeasure is to freshly prepare a lysozyme solution, centrifuge it 10 min at 50,000 × *g*, and use the resulting supernatant for microcrystallization. In addition, it is important to ensure that the pH meter is properly calibrated and that buffers are prepared at the accurate pH.

## Basic Protocol 2 MICROCRYSTALLIZATION OF COPPER‐CONTAINING NITRITE REDUCTASE

Copper‐containing nitrite reductase (CuNiR) catalyzes the reduction of NO_2_^–^ to nitric oxide (NO_2_^–^ + 2H^+^ + e^–^ → NO + H_2_O), which is a key step in microbial denitrification in the global nitrogen cycle. Traditionally, structural analyses of CuNiR using SRX have encountered difficulties because X‐ray photoreduction induces structural changes to the metal centers and the enzyme–substrate complex. However, we used the photoreduction induced by SRX to initiate a catalytic reaction of CuNiR and to trap enzymatically produced intermediary structures. Furthermore, we visualized intact and oxidized CuNiR structures using SFX. By comparing the SRX and SFX structures, we successfully revealed protein motions during catalysis and obtained new insights into the CuNiR reaction mechanism (Fukuda, Tse, Nakane et al., 2016; Fukuda, Tse, Suzuki et al., 2016).

Microcrystals of CuNiR were obtained by the rotational seed crystallization technique, which is considerably different from the microcrystallization procedure used for the preparation of lysozyme crystals, as described above. In our experience, many protein microcrystals cannot be prepared homogeneously using the protocol for the preparation of lysozyme microcrystals. Therefore, the technique introduced here is recommended as a primary option. We have succeeded in using this technique for several proteins, including bacteriorhodopsin in bicelles (Hanashima et al., 2021; Nakane, Hanashima et al., 2016).

### Materials

Purified CuNiR from *Alcaligenes faecalis* strain S‐6 (Fukuda, Tse, Nakane et al., 2016)
1.0 M Sodium acetate trihydrate, pH 4.0 (Hampton Research, HR2‐933‐05)
PEG 4000, 50% (w/v) (Hampton Research, HR2‐529)

24‐well hanging drop crystallization plate
Microtubes, 1.5 µl (Eppendorf)
UR‐20P Handy sonic (Tomy)
High‐speed refrigerated microcentrifuge (Tomy)
Centrifuge tubes, 15 ml (Falcon)
RT‐50 rotator (Taitec)
CellTrics filters, 100, 50, and 30 µm (Sysmex)
High‐performance microscope (Hirox KH‐8700)
Slide glass and cover glass

#### Rotational seeding microcrystallization of CuNiR

**Figure 2:**
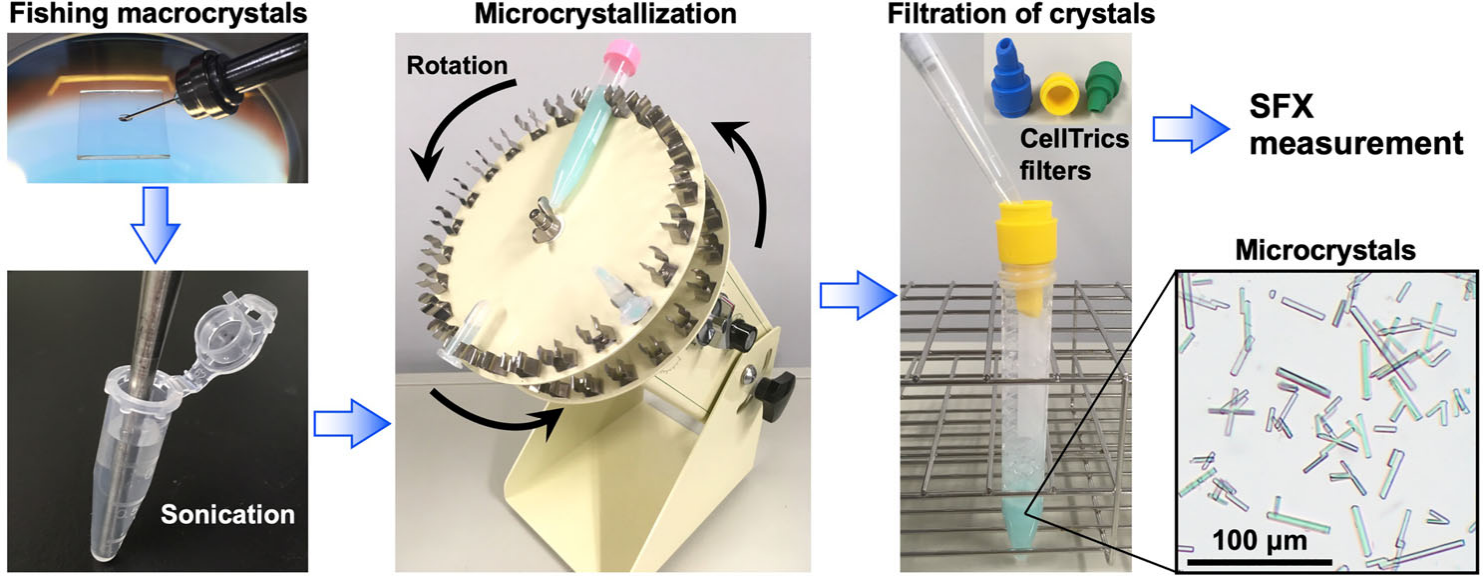
Microcrystallization scheme for CuNiR using the rotational seeding technique.

The protocol consists of three steps: (i) preparation of macrocrystals; (ii) preparation of nanoseeds; and (iii) microcrystallization using the rotational seed crystallization technique (Fig. 2).

1 Crystallize CuNiR, purified as described previously (Fukuda, Tse, Nakane et al., 2016), by the hanging‐drop vapor‐diffusion method at 293 K with a 24‐well hanging drop crystallization plate. Drops contain a 1:1 mixture of the purified protein solution (50 mg/ml) and a reservoir solution composed of 100 mM sodium acetate (pH 4.0) and 7% (w/v) PEG 4000. Allow multiple macrocrystals to grow over a few days; they typically reach their maximum size within 1 week. Use the grown crystals for the subsequent nanoseeding step.
2 Select single crystals measuring ∼1000 µm × ∼300 µm × ∼200 µm and place them into a 1.5‐ml microtube containing 1 ml of 100 mM sodium acetate (pH 4.0) and 10% (w/v) PEG 4000. The concentration of PEG 4000 is 3% higher than that used in step 1 for crystal stabilization. The CuNiR always crystallizes in space group *P*2_1_2_1_2_1_. Thus, multiple crystals can be taken from the crystallization drops for preparing the nanoseeds. However, depending on the type of protein, crystals of different space groups may be produced in a single drop. In this case, by fishing out only one crystal for seed preparation, uniform space group microcrystallization should be achieved.
3 Prepare the nanoseed solution by sonicating the macrocrystals in the microtube with the handy sonic for 5 min. Do not regard the softening of CuNiR crystals or deterioration of seed morphology during continuous heating for 5 min as a significant concern. However, avoid raising the sample temperature excessively, as this may cause the crystals to melt.
4 Centrifuge the sonicated sample 1 s at 15,000 × *g* to remove larger, incompletely fragmented crystals as pellets and to collect the smaller crystals in the supernatant. Repeat the procedure two additional times and use the resulting solution as nanoseeds. Depending on the type of protein, due to the large specific gravity of the crystals, the nanoseeds may easily sink to the bottom of the microtube following centrifugation. In this case, reduce the centrifugal force to avoid seed loss.
5 Prepare microcrystals by the rotational seeding crystallization technique. In a 15‐ml centrifuge tube, mix 500 µl of the protein solution (50 mg/ml) with 10 ml of precipitant solution containing 100 mM sodium acetate (pH 4.0) and 12% (w/v) PEG 4000, and add 20 µl of the nanoseed solution. Perform preliminary crystallization experiments on a smaller scale to optimize conditions.
6 Place the centrifuge tube on an RT‐50 rotator at a speed of 30 rpm for 4 days at 293 K to obtain microcrystals. The seeding can quickly initiate crystal growth of uniform space groups. Rotation prevents the crystals from growing to an excessively large size.
7 Filter the microcrystal solution sequentially through 100‐, 50‐, and 30‐µm CellTrics filters. Check the sizes of the filtered microcrystals by observing under a high‐performance microscope. The size distributions of CuNiR microcrystals are usually between 10 and 80 µm. Because the CuNiR microcrystals are typically rod‐shaped, they may pass through the 30‐µm filter when oriented longitudinally, even if one dimension exceeds 30 µm. Variations in crystal size do not pose a problem for SFX data collection.

## Basic Protocol 3 TIME‐RESOLVED SERIAL FEMTOSECOND CRYSTALLOGRAPHY

Time‐resolved serial femtosecond crystallography (TR‐SFX) is a powerful tool for visualizing the structural dynamics of proteins at work. In particular, TR‐SFX in conjunction with photosensitive caged compounds has enabled the investigation of the dynamics of various proteins, including enzymes. Here, nitric oxide (NO) reductase isolated from the fungus *Fusarium oxysporum* (P450nor), a simple heme enzyme, will be used, and the procedure for capturing an initial intermediate (NO‐bound state) during its enzymatic reaction (2NO + NADH + H^+^ → N_2_O + H_2_O) will be described as a model study (Tosha et al., 2017).

### Materials

pRSET‐C–P450nor expression plasmid (*Fusarium oxysporum* gene)
*Escherichia coli* BL21(DE3) competent cells
LB medium powder
Terrific Broth powder
Isopropyl β‐D‐1‐thiogalactopyranoside (IPTG)
Potassium phosphate, monobasic/dibasic
Glycerol
Bis‐Tris buffer
Bis‐Tris propane
PEG 10000, 50% (w/v) (Hampton Research, HR2‐607)
Ammonium acetate
Caged nitric oxide donor (caged‐NO): *N,N'*‐bis(carboxymethyl)‐*N,N'*‐dinitroso‐*p*‐phenylenediamine sodium salt (BNN 5 Na)
β‐Nicotinamide adenine dinucleotide, reduced form (NADH), disodium salt
Hydroxyethyl cellulose (Sigma, 09368)

Probe sonicator
Dialysis tubing
DEAE‐FF anion‐exchange columns (Cytiva)
Sitting‐drop vapor‐diffusion plates
PCR tubes
Red LED safelight (for handling caged‐NO)
Hamilton gas‐tight syringes
LCP mixer device (Art Robbins Instruments)
LCP‐type microextrusion injector with 75‐µm sapphire nozzle (custom at SACLA)
Optical parametric oscillator (OPO, NT230, EKSPLA)
Pulse generator (DG645, Stanford Research Systems)

#### Purification of P450nor

1 Use a pRSET‐C vector encoding P450nor from the fungus *Fusarium oxysporum* to express the recombinant enzyme.
2 Transform the vector into *Escherichia coli* BL21(DE3) cells and express the recombinant P450nor.
3 Culture the transformed cells in 100 ml of LB medium at 37°C overnight. Inoculate the pre‐cultured cells into 1 l of Terrific Broth and grow at 30°C. After 4 h of culturing, add IPTG to obtain a final concentration of 0.05 mM and continue culturing overnight at 30°C.
4 Harvest the cells by centrifugation at 8000 × *g* for 10 min at 4°C. Resuspend the cell pellet in 5 mM potassium phosphate buffer (pH 8.0) containing lysozyme, and lyse the cells by sonication for 15 min.
5 Remove insoluble material by ultracentrifugation at 165,000 × *g* for 30 min. Dialyze the supernatant (soluble fraction) against 5 mM potassium phosphate buffer (pH 8.0).
6 Load the dialyzed soluble fraction onto an anion exchange column pre‐equilibrated with 5 mM potassium phosphate buffer (pH 8.0). Wash the column with the same buffer to remove unbound proteins and elute the target protein using a linear gradient of 5–50 mM potassium phosphate.
7 Dialyze the reddish fractions containing the target protein against 10 mM potassium phosphate buffer (pH 6.0) containing 10% (v/v) glycerol.
8 Load the dialyzed sample onto a second DEAE‐FF column pre‐equilibrated with 10 mM potassium phosphate buffer (pH 6.0) containing 10% (v/v) glycerol. Wash unbound material with the same buffer and elute the target protein using a 10–20 mM potassium phosphate gradient.
9 Exchange the buffer of the purified protein fractions (with *A*_413_/*A*_280_ > 1.8) that showed a single band on SDS‐PAGE into 20 mM potassium phosphate buffer (pH 6.0) containing 10% (v/v) glycerol. This selection based on SDS‐PAGE purity was critical, as fractions with minor contaminants did not yield reproducible crystals.

#### Crystallization of P450nor

10 Crystallize purified P450nor, as described previously (Shimizu et al., 2000), using the sitting‐drop vapor diffusion method at 293 K. Prepare crystallization drops by mixing 1 µl of 25 mg/ml P450nor solution with 1 µl of reservoir solution containing 100 mM Bis‐Tris buffer (pH 5.6–6.8), 18%–34% (w/v) PEG 10000, and 150 mM ammonium acetate. Equilibrate each drop against 500 µl of reservoir solution overnight. Crystals typically appeared within 12 h, and those grown for 24 h or longer were used for subsequent microseeding.
11 Obtain plate‐like single crystals measuring ∼500 µm × ∼100 µm × ∼50 µm by microseeding under the same crystallization conditions as in step 10.
12 Prepare microcrystals using a batch method with microseeding. In a PCR tube, mix 50 µl of 5.0 mg/ml P450nor solution with 50 µl of crystallization mother liquor containing 100 mM Bis‐Tris propane (pH 8.5), 34%–38% (w/v) PEG 10000, and 150 mM ammonium acetate, a condition optimized to suppress overgrowth and reproducibly yield microcrystals suitable for SFX. Crush 10–20 pieces of the plate‐like single crystals in ∼100 µl of the same mother liquor to generate microseeds. Add the microseeds to the crystallization mixture. Incubate the tubes at 293 K for 1 day to obtain microcrystals with a length of 20–50 µm and a thickness of less than 10 µm.

#### Section 3: Preparation of TR‐SFX samples soaked with a caged compound

Prepare P450nor microcrystals soaked with a caged nitric oxide donor (caged‐NO) and NADH for TR‐SFX analysis. Use *N,N'*‐bis‐(carboxymethyl)‐*N,N'*‐dinitroso‐*p*‐phenylenediamine as the caged‐NO compound, which releases two NO molecules upon UV illumination (Namiki et al., 1997). Perform all procedures under red light to prevent photodecomposition of the caged compound.

13 Harvest the P450nor microcrystals by centrifugation at 8000 × *g* for 1 min at 273 K. Resuspend the pellet in the crystallization solution containing 100 mM Bis‐Tris propane (pH 8.5), 38% (w/v) PEG 10000, and 150 mM ammonium acetate, supplemented with 20 mM caged‐NO and 140 mM NADH.
14 Incubate the microcrystal suspension at ambient temperature for at least 10 min.
15 Independently prepare an SFX carrying medium (Sugahara et al., 2017). Prepare 40% (w/v) hydroxyethyl cellulose in 100 mM Bis‐Tris propane (pH 8.5), 36% (w/v) PEG 10000, 150 mM ammonium acetate, and 10% (v/v) glycerol as a cellulose stock solution. Dilute the cellulose stock solution with 100 mM Bis‐Tris propane (pH 8.5), 36% (w/v) PEG 10,000, 150 mM ammonium acetate containing 65 mM caged‐NO, and 155 mM NADH to give a 32% (w/v) hydroxyethyl cellulose solution containing 13 mM caged‐NO and 31 mM NADH as a cellulose‐based carrying medium. The cellulose stock solution and the dilution solution are mixed using two gas‐tight syringes connected by a coupler. These solutions must be mixed for a minimum of 200 times using an LCP mixer device (ARI) to obtain a homogeneous carrying medium.
16 Concentrate the microcrystals by centrifugation and mix 10 µl of the concentrated microcrystal slurry with 90 µl of the carrying medium, 32% (w/v) hydroxyethyl cellulose solution containing 13 mM caged‐NO and 31 mM NADH [final concentration: 29% hydroxyethyl cellulose (w/v)], 14 mM caged‐NO, and 42 mM NADH (see step 24).

#### TR‐SFX of P450nor triggered by the caged compound

**Figure 3:**
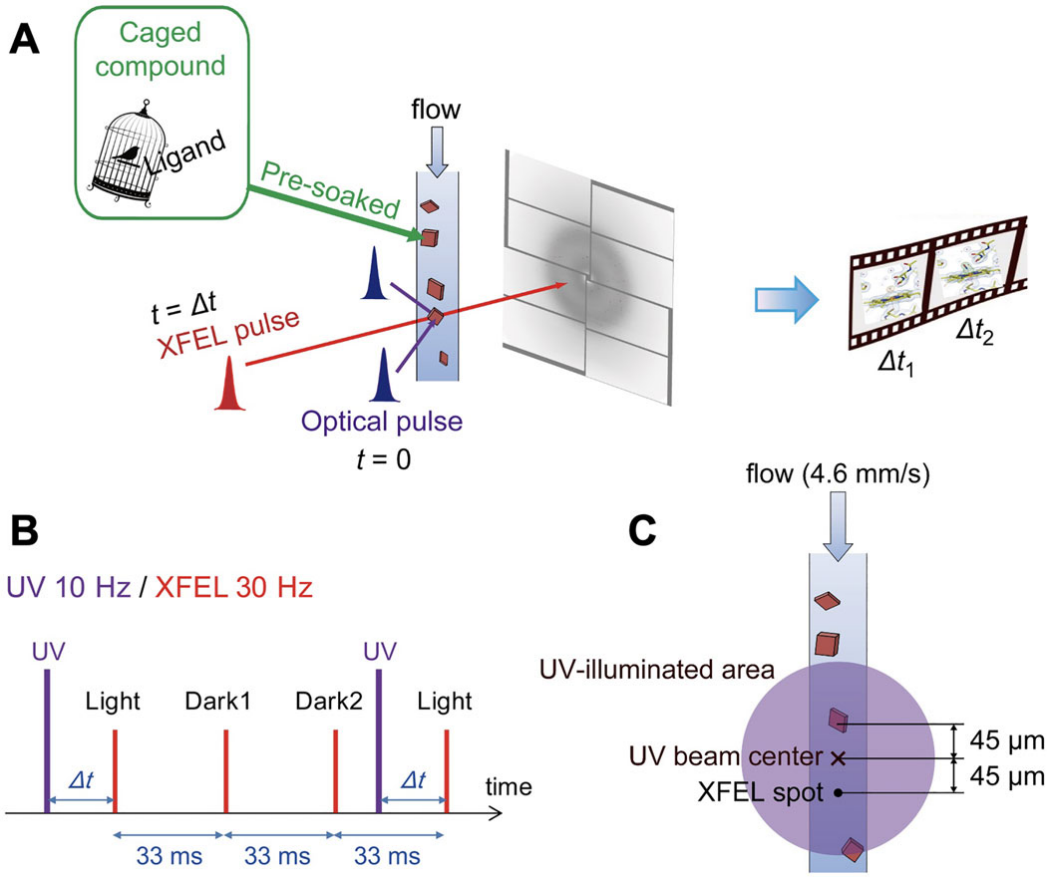
Time‐resolved serial femtosecond crystallography (TR‐SFX). (**A**) TR‐SFX experimental scheme with a caged compound for fungal nitric oxide reductase (P450nor). (**B**) Timing chart of UV and XFEL illumination. (**C**) Spatial alignment for UV illumination of the sample stream.

This TR‐SFX method uses optical pulse illumination to photolyze a caged compound at time zero (*t* = 0), followed by XFEL pulse illumination for diffraction data collection at a defined delay time (*t* = *Δt*) (Fig. 3A). By collecting structural snapshots at different *Δt* values, a molecular movie that captures the dynamics of protein function is generated. The following procedure describes the method implemented at SACLA.

17 Choose the OPO as the reaction trigger light source. The output pulse duration of the OPO is 6 ns, and its wavelength is tunable from 300 to 2000 nm. Set the wavelength to 308 nm for release of the caged‐NO. Use the UV illumination system in a nearly counter‐propagating geometry (Kubo et al., 2017), which enables UV illumination of the sample from two directions to increase the excitation efficiency. The suitable UV pulse energy depends on the quantum yield of caged compounds (see step 25). The UV focal size is Ø250 µm at the sample point.
18 Set the UV pulse repetition rate to 10 Hz, which is one‐third of that of XFEL (30 Hz) (*see* step 26). Under this condition, a diffraction image from a UV‐illuminated crystal (“Light”), followed by two images from non‐UV‐illuminated crystals (“Dark1” and “Dark2”), are obtained sequentially (Fig. 3B). The “Dark” images are used as the reference for capturing the protein dynamics by calculating *F*_o_ (“Light”) − *F*_o_ (“Dark”) difference Fourier maps.
19 Set the delay time (*Δt*) between UV and XFEL pulses, which is controlled by the pulse generator. Note that a timing jitter is <1 ns for *Δt* < 16.7 ms, but ∼0.1 ms for *Δt* > 16.7 ms at SACLA. For the initial intermediate analysis of P450nor, the *Δt* value is 20 ms.
20 Deliver the P450nor microcrystals with the carrier medium using an LCP injector with a 75‐µm nozzle at a flow rate of 4.6 mm/s (1.2 µl/min). Maintain the temperature inside the injector at 293 K. Adjust the suction force of the vacuum device below the injector nozzle to stabilize the sample stream (see step 27).
21 Align the UV beam center 45 µm upstream from the 3 × 3 µm^2^ XFEL spot (see step 28) (Fig. 3C). At a flow rate of ∼4.6 mm/s, the sample travels ∼90 µm in 20 ms, whereas the pump–probe delay is defined electronically rather than by crystal displacement. Accordingly, use a broadened illumination centered at 45 µm upstream that spans from the XFEL interaction point (0 µm) to ∼90 µm upstream, ensuring that pumped crystals provide data at *Δt* = 20 ms, regardless of flow fluctuations.
22 Collect the diffraction images. Use the data processing pipeline (Nakane, Joti et al., 2016) based on Cheetah (Barty et al., 2014) for real‐time feedback during the beam time, hit finding, and sorting of “Light” and “Dark” images.
23 Repeat the diffraction measurements with different *Δt* values of interest to produce a molecular movie.

#### Notes for TR‐SFX of P450nor

24 Use a final concentration of 14 mM caged‐NO, which is sufficient for intermediate structural analysis given the high quantum yield of caged‐NO (1.4 upon 308‐nm excitation) (Tosha et al., 2017) and the 16 mM concentration of P450nor in the crystal. Prepare the sample to contain 42 mM NADH, the highest concentration achievable under the present experimental conditions.
25 To enhance photolysis efficiency, increase the UV pulse energy as needed. However, to avoid UV‐induced protein damage, reduce the UV pulse energy at the sample point to approximately 0.8 nJ/µm² (combined energy from two directions) for P450nor.
26 Set the output of the OPO laser to a submultiple of 30 Hz, typically 10 or 15 Hz, for TR‐SFX at SACLA. At a pump repetition rate of 15 Hz, diffraction images alternate between Light and Dark. At a pump repetition rate of 10 Hz, each Light image is followed by two Dark images, yielding a Light–Dark1–Dark2 sequence. Divert approximately 5% of the UV beam near the OPO head and detect the voltage signal with a photodiode to tag the corresponding image as “Light” during data acquisition.
27 Maintain a stable, continuous sample stream to ensure proper sample exchange between UV‐illuminated and non‐illuminated crystals. Complete the exchange within 33.3 ms at a 15‐Hz UV pulse repetition rate, and within 33.3 ms (“Dark1”) or 66.6 ms (“Dark2”) at 10 Hz. Use the 10 Hz setting to allow sufficient time for exchange. If sample exchange cannot be completed within the required window—as was the case under the current experimental conditions—use “Dark2” images as the reference in difference Fourier map calculations. Alternatively, increase the sample flow rate to improve exchange efficiency, while noting that this increases sample consumption.
28 At a flow rate of ∼4.6 mm/s, the sample moves ∼90 µm downstream in 20 ms. Design the UV illumination area to cover ∼90 µm upstream of the XFEL spot and include the XFEL spot itself (Fig. 3C). Ensure spatial overlap between the UV and XFEL beams to maintain the integrity of the “Light” data and minimize the effect of sample stream fluctuations. If the UV‐illuminated region is positioned too far from the XFEL spot, accurately determine the sample flow rate to align the illuminated crystal with the XFEL beam at *Δt* = 20 ms.

## Commentary

### Critical Parameters

#### Lysozyme microcrystallization

Obtaining a monodisperse suspension of lysozyme crystals hinges on three tightly coupled variables—pH, temperature, and mixing rate—as well as other factors such as ionic strength (precise NaCl concentrations), PEG polydispersity, and protein concentration. Setting the precipitant buffer to the appropriate pH is essential; even a 0.2‐unit deviation in pH induces aggregation, polydispersity, or slow nucleation. Temperature governs the final crystal length; holding the thermomixer at 17°C for the full 30‐min pre‐equilibration and the subsequent 10‐min post‐mix incubation is critical for the targeted 5‐µm long axis. Temperature deviations of ±2°C can shift the crystal size distribution by 1–2 µm, complicating density adjustment, and such size changes do not necessarily follow a strict linear rule. A rapid, one‐stroke combination of the precipitant with the protein solution ensures instantaneous nucleation; hesitation of more than a few seconds allows local supersaturation gradients that seed oversized crystals. After centrifugation, resuspension in fresh harvest buffer must be gentle yet thorough; incomplete resuspension traps precipitate pockets that later clog the injector filter, whereas vigorous vortexing fragments crystals and increases mosaicity.

#### Rotational seed crystallization of CuNiR

Homogeneity in this protocol is governed primarily by the quality of the nanoseeds and the rotational speed. Sufficient sonication of macrocrystals is necessary to obtain seeds small enough for effective nucleation; insufficient sonication leaves larger shards that act as competing nuclei and broaden the size distribution. Care should be taken to avoid overheating during sonication, for example, by applying pulsed bursts and placing the tube on ice temporarily, since excessive heating can partly dissolve seeds and lower nucleation efficiency, whereas prolonged chilling may also disturb the crystallization conditions. Immediate triple clarification at 15,000 × *g* prevents sedimentation of the heavier copper‐rich debris, which otherwise biases seed concentration toward early aliquots. The rotational speed of 30 rpm is not arbitrary: slower rotation allows macrocrystal growth on the tube wall, while faster rotation generates shear that fragments nascent microcrystals. The PEG concentration must be within ±0.5% (w/v) of the stated values; a higher PEG concentration accelerates nucleation but yields needle clusters, whereas a lower PEG concentration reduces the overall crystal yield. When handling highly redox‐ or photosensitive proteins, dissolved O_2_ must be tightly controlled. Use freshly degassed buffers and reducing agents to set aerobic or anaerobic conditions where necessary. All manipulations should be performed under dim light, or, when feasible, in complete darkness.

#### TR‐SFX with P450nor

Reproducible capture of the NO‐bound intermediate rests on maintaining strict control over photochemistry and fluid dynamics. The final concentrations of 14 mM caged NO and 42 mM NADH were chosen to ensure complete substrate delivery without exceeding the solubility limit; lowering the concentration of caged NO reduces the population of the intermediate due to insufficient substrate availability, whereas raising it too high attenuates UV penetration into the crystal interior, diminishing intermediate formation. All steps from soaking to injector loading must occur under red light; exposure to ambient light partially photolyzes the caged compound and diminishes the UV‐triggered population difference. Homogenizing the 32% hydroxyethyl cellulose carrier by a minimum of 200 reciprocations through the LCP mixer eliminates viscosity gradients that destabilize the 75‐µm jet. Flow rate and UV‐to‐XFEL delay are interdependent: at 4.6 mm s^−1^, a 20‐ms delay translates to ∼90‐µm travel; altering the delay without recalibrating the UV focal position will misregister the excited volume. Finally, the stability of the continuous jet is non‐negotiable; any pulsation in the flow can allow UV‐illuminated material to persist into a “Dark” frame, degrading the quality of the difference maps. Real‐time monitoring of the hit rate and mosaicity is therefore indispensable for early detection of subtle stream instabilities.

### Troubleshooting

See Table 1 for a list of problems, causes, and solutions.

**Table 1:** Troubleshooting Guide for Microcrystallization and Serial Femtosecond Crystallography

Problem	Possible cause	Solution
Microcrystals obtained too small	Low purity of the protein sample	Improve sample purity by optimizing purification conditions.
	Presence of aggregates	Remove aggregates by ultracentrifugation.
	Protein concentration too high	Dilute the protein solution.
	Suboptimal nucleation or growth conditions	Reduce the amount (density) of added seed crystals. Optimize crystallization conditions by fine‐tuning pH and precipitant concentration. Use low‐speed centrifugation to selectively precipitate and recover larger crystals while leaving smaller ones in suspension.
Microcrystals obtained too large	Protein concentration relatively low	Concentrate the protein solution.
	Suboptimal nucleation or growth conditions	Increase the amount (density) of added seed crystals. Optimize crystallization conditions such as pH, precipitant concentration, and temperature. Remove large crystals by passing the suspension through appropriately sized CellTrics filters once or multiple times.
Microcrystals sticking together	Low purity of the protein sample	Improve sample purity by optimizing purification conditions.
	Denatured protein	Re‐screen the crystallization conditions or modify the protein expression system.
No crystals observed	Unstable or impure protein	Check sample quality by sodium dodecyl sulfate–polyacrylamide gel electrophoresis (SDS‐PAGE) and dynamic light scattering (DLS).
	Crystallization conditions unsuitable	Explore broader crystallization screens.
No structural change detected after UV illumination (TR‐SFX)	Low photolysis efficiency of the caged substrate (quantum yield too low at chosen wavelength)	Use a caged compound with a higher quantum yield or verify the photolysis spectrum; adjust the excitation wavelength accordingly.
	Insufficient pump–laser fluence at the crystal stream	Increase the UV pulse energy or improve beam alignment/focus at the interaction point; confirm fluence using a power meter.
	Crystal dimensions too large, causing incomplete penetration of UV light	Reduce crystal size or decrease sample stream thickness.
	Incorrect pump–probe timing (*Δt* incorrectly set)	Re‐optimize *Δt* based on reaction kinetics.
	Sample heterogeneity or partial occupancy of substrate	Confirm substrate occupancy by spectroscopy/SDS‐PAGE; increase soaking concentration or incubation time.

### Understanding Results

Successful implementation of the three protocols can be evaluated at several checkpoints, each providing quantitative and qualitative benchmarks confirming that the experiment is progressing toward high‐quality structural information. For lysozyme microcrystallization, the immediate visual cue of success is the appearance of uniformly sized crystals within a day of mixing; a high‐performance microscope should reveal sharp edges with minimal clustering (Fig. 1). The crystal density, when measured with a hemocytometer, should fall between 1 × 10⁸ and 2 × 10⁸ crystals/ml. Lower densities correlate with extended data collection times, whereas higher densities increase multihit diffraction patterns that reduce indexing rates. Diffraction snapshots collected during a brief test run should exhibit a hit rate (percentage of detector frames with crystal diffraction patterns) of approximately 20%–50%, demonstrating that the crystal size distribution and density are adequate for routine detector calibration and geometry refinement.

For the rotational seeding strategy applied to CuNiR, visual inspection should reveal a heterogeneous population of needle‐ or rod‐shaped crystals ranging from 10 to 80 µm that stay in suspension under gentle agitation, reflecting the continuous nucleation that accompanies slow rotation (Fig. 2). During sample filtration, progressive passage through 100‐, 50‐, and 30‐µm meshes removes larger outliers without appreciable loss of smaller crystals. Static SFX data sets derived from these crystals routinely achieve an overall resolution of 1.5–2.0 Å. When rotational seeding has a homogenized space group and an effective mosaic spread, the resulting electron density maps often show continuous side‐chain density even for surface residues.

TR‐SFX of P450nor provides two layers of validation: the integrity of the light–dark pairing scheme and the chemical plausibility of the refined intermediates. In real time, the hit finder output should be continuously monitored to ensure that data acquisition is proceeding as intended. After full integration and scaling, the omit map for the heme pocket should reveal a positive difference density at the distal side of the iron, consistent with NO binding (Fig. 3).

### Time Considerations

Successful application of these methods requires careful scheduling that spans laboratory preparation, crystal growth, and beam‐time execution. Solution preparation for the reference lysozyme sample can be performed the day before use; once the buffers are equilibrated, microcrystals of the desired size form within an hour and are ready for density measurements, allowing an experienced user to complete the entire procedure in a single working day.

Producing homogeneous microcrystals of CuNiR demands a longer lead time: macrocrystals typically reach a suitable size in 3–7 days, nanoseed preparation takes less than an hour, and rotational seeding requires a further 4 days to yield a stable microcrystal slurry.

Expression and purification of the heme enzyme P450nor can be accomplished in about 3 working days—overnight preculture, 1 day for high‐density growth and induction, and 1 day for chromatographic purification—followed by 1–2 days for crystallization and microseeding to obtain plate‐like seeds and sub‐50 µm microcrystals. Soaking with a caged substrate and embedding the crystals in the cellulose carrier adds roughly half a day, provided all stock solutions are prepared in advance. Because TR‐SFX experiments depend on tightly allocated XFEL beam time, all samples should be finalized and quality checked at least 24 h before the scheduled shift to accommodate shipping or late‐night facility access. A typical 12‐h shift at 30 Hz is sufficient to collect about 10^5^ indexed patterns from a single well‐optimized sample; additional shifts are required to gather datasets at multiple delay points. On‐the‐fly data reduction with the Cheetah‐based pipeline provides immediate feedback, enabling laser‐timing or flow‐rate adjustments within minutes. Full integration, merging, and preliminary difference Fourier analysis are generally completed within a week after beam time, whereas atomic model refinement and movie assembly usually require an additional 1–2 weeks, depending on the complexity of the intermediates and the number of time points collected.

### Conclusion

The protocols described here provide a practical framework for tracking protein motions at room temperature using SFX with XFELs. By establishing workflows for lysozyme microcrystallization, rotational seeding of copper‐containing nitrite reductase, and time‐resolved studies of nitric‐oxide reductase, we demonstrate strategies that are broadly adaptable to diverse protein targets. Together, these methods enable researchers to prepare high‐quality microcrystals, optimize data collection, and capture transient structural intermediates at near‐atomic resolution. With continued advances in XFEL instrumentation and methodology, such approaches will facilitate increasingly detailed molecular movies of enzymatic reactions, offering new insights into protein structure–function relationships.

### Author Contributions

**Eiichi Mizohata**: Conceptualization; data curation; formal analysis; funding acquisition; investigation; methodology; visualization; writing original draft; writing review and editing. **Eriko Nango**: Conceptualization; data curation; formal analysis; investigation; methodology; visualization; writing original draft; writing review and editing. **Takehiko Tosha**: Conceptualization; data curation; formal analysis; investigation; methodology; visualization; writing original draft; writing review and editing. **So Iwata**: Funding acquisition; project administration; supervision. **Minoru Kubo**: Conceptualization; data curation; formal analysis; investigation; methodology; visualization; writing original draft; writing review and editing.

### Conflict of Interest

The authors declare no conflict of interest.

## Data Availability

The data that support the findings of this study are available from the corresponding author upon reasonable request.
